# Assessment of Vcheck^®^ analyzer for rapid progesterone concentration measurement including recommendations for achieving the optimal breeding time in bitches

**DOI:** 10.14202/vetworld.2024.427-433

**Published:** 2024-02-20

**Authors:** Supphathat Wutthiwitthayaphon, Thanikran Suwannachote, Saengtawan Arayatham, Wisut Prasitsuwan, Sakchai Ruenphet

**Affiliations:** 1 Department Immunology and Virology, Veterinary Medicine Faculty, Mahanakorn University of Technology, 140 Cheum-Sampan Rd. Nong Chock, Bangkok, Thailand; 2 Clinic for Small Domestic Animals and Radiology, Veterinary Medicine Faculty, Mahanakorn University of Technology, 140 Cheum-Sampan Rd. Nong Chock, Bangkok, Thailand; 3 Master of Science Program in Animal Biotechnology, Veterinary Medicine Faculty, Mahanakorn University of Technology, 140 Cheum-Sampan Rd. Nong Chock, Bangkok, Thailand; 4 Clinic of Obstetrics, Gynecology and Animal Reproduction, Veterinary Medicine Faculty, Mahanakorn University of Technology, 140 Cheum-Sampan Rd. Nong Chock, Bangkok, Thailand

**Keywords:** chemiluminescent microparticle immunoassay, optimal breeding time, progesterone, Vcheck^®^

## Abstract

**Background and Aim:**

Serum progesterone concentration plays critical role in determining the optimal breeding time in bitches and diagnosing reproductive-related issues. This study aimed to conduct a comparative analysis of serum progesterone results obtained from commercial point-of-care immunological analyzers, namely, Vcheck^®^, with those obtained using chemiluminescent microparticle immunoassay (CMIA). Our overarching goal was to evaluate these analyzers’ accuracy and establish standardized guidelines for optimal breeding timing.

**Materials and Methods:**

Ninety-four serum samples from bitches were analyzed using the Vcheck^®^ analyzer and compared with CMIA. Thorough documentation included the mean, standard deviation, 95% confidence interval (CI), and minimum and maximum values of serum progesterone concentrations. Furthermore, Pearson’s correlation coefficient, Lin’s concordance correlation coefficient, and the bias correction factor were meticulously recorded.

**Results:**

The mean progesterone concentration measured using the Vcheck^®^ analyzer was significantly lower than that measured using CMIA, with a mean difference of 1.26 ng/mL of serum. The Bias correction factor was 0.935, which was nearly 1.00, indicating that the line of best-fit was on the perfect line of agreement, providing insight into the measurement accuracy. Pearson’s correlation coefficient, a measure of precision, was also close to 1 (0.939), confirming the reliability of the data. Furthermore, Lin’s concordance correlation coefficient was 0.877, indicating a fair overall agreement between the Vcheck^®^ and CMIA methods. These results support the validity of the Vcheck^®^ analyzer’s results. The present study was developed by aligning with established CMIA guidelines and adapting them using the range and 95% CI derived from each set of results, ensuring a standardized and rigorous approach.

**Conclusion:**

The Vcheck^®^ analyzer provides a rapid assessment of serum progesterone concentration in bitches, with results comparable to those measured using the CMIA technique. However, when considering the use of the Vcheck^®^ analyzer, it is recommended that the results should be interpreted carefully and the interpretation guidelines should be followed. In conclusion, Vcheck^®^ provides a reliable and convenient method for veterinarian practitioners to measure canine progesterone levels in a clinical/hospital setting.

## Introduction

The optimal breeding time in bitches requires an assessment of serum progesterone concentrations [1]. However, this evaluation serves multiple essential purposes, including identifying reproductive irregularities, such as hypoluteoidism [2], and confirming luteolysis before parturition [3–5]. Notably, a characteristic increase in serum progesterone concentrations during the estrus period often exceeding 1 ng/mL, indicates significant hormonal changes. In bitches, ovulation typically occurs 36–50 h after the luteinizing hormone (LH) peak [6], which correlates with serum progesterone concentrations of approximately 2.02 0.18 ng/mL [7]. These concentrations then escalate to a range of 4.00–10.00 ng/mL on the day of ovulation [8], indicating a significant hormonal shift and the onset of ovulation. Intriguingly, despite this range, Seefeldt *et al*. [9], Marseloo *et al*. [10], and Mir *et al*. [11] suggested a serum progesterone concentration of 5.00–8.00 ng/mL, introducing contrasting perspectives on determining the ovulation.

Various techniques, such as radioimmunoassay (RIA) [12, 13], liquid chromatography-tandem mass spectrometry (LC-MS) [14, 15], and chemiluminescence immunoassay (CLIA) [14, 16], are used to measure serum progesterone concentration in veterinary medicine. An accurate and reliable alternative is the CLIA method, adept at assaying serial blood samples with guaranteed safety, speed, accuracy, and repeatability [17]. CLIA addresses the drawbacks associated with RIA and enzyme immunosorbent assay and serves as a robust solution for precise serum progesterone monitoring, aiding in accurate ovulation prediction and confirmation. Moreover, recent advances, such as point-of-care analyzers, have transformed the measurement of progesterone. These advancements, including rapid fluorescence immunochromatography assay, surface plasmon field-enhanced fluorescence spectroscopy, lateral flow immunochromatography, and competitive enzyme-linked fluorescence assay [18–21], enhance serum progesterone monitoring, ultimately improving veterinary practice in managing reproductive processes.

However, differences in serum progesterone levels are due to the use of different laboratory techniques and variations between bitches. As a result, the precise identification of the ideal mating period necessitates the collection of multiple consecutive blood samples during both the proestrus and estrus phases, which can then be compared with established gold standards or reference laboratory procedures. This investigation involves a comparative analysis of serum progesterone findings derived from a commercial point-of-care analyzer, namely Vcheck^®^, in contrast to those acquired through chemiluminescent microparticle immunoassay (CMIA) using the same serum samples.

## Materials and Methods

### Ethical approval and informed consent

The study was approved by Animal Research Ethics Committee of the Faculty of Veterinary Medicine at Mahanakorn University of Technology, Thailand (approval number ACUC-MUT-2020/006). The owners of the bitches expressed consent to participate in the research by signing an official document.

### Study period and location

Blood samples were obtained for analytical purposes from August 2020 to July 2023. This acquisition took place at two locations: the Small Animal Teaching Hospital at the Faculty of Veterinary Medicine, Mahanakorn University of Technology, Thailand, and Vet Home Polyclinic in Bangkok, Thailand.

### Sample collection and progesterone measurement

Ninety-four serum samples were collected from bitches of various ages and breeds, including American bullies, English bulldogs, French bulldogs, Shetland sheepdogs, miniature American shepherds, Cavalier King Charles spaniels, Chihuahuas, Pomeranians, Chow Chows, Akitas, and Pugs. To ensure a comprehensive analysis, all bitches underwent both progesterone concentration determination and vaginal cytology examination, aligning with the study’s multifaceted approach. The aim of this dual approach was to provide a comprehensive understanding of hormonal changes and reproductive stages. The criteria for sampling were defined by vaginal cytology; specifically, 70% cornified epithelial cells were observed on the 1^st^ day, indicating late proestrus. Consequently, a blood sample was collected for progesterone determination to capture hormonal changes during this transitional phase. Subsequently, additional samples were collected on the day when vaginal cytology showed 90% cornified epithelial cells, signifying the onset of the estrus period. This transition from late proestrus to estrus allowed a comprehensive assessment of progesterone fluctuations in different reproductive cycle stages. Two aliquots of serum were meticulously prepared for each bitch sample. One aliquot was immediately used to assess progesterone concentration using CMIA, ensuring immediate measurements for real-time analysis. An Architect i2000SR Immunoassay Analyzer (Abbott Laboratories, Illinois, USA) along with the Architect Progesterone Reagent (Abbott Laboratories, Illinois, USA) was used for this process. Simultaneously, the second aliquot was judiciously stored at 20°C until it was required for assessment. Strategic storage was implemented to facilitate subsequent evaluations performed using commercial point-of-care analyzers, specifically the Bionote V200 analyzer (Bionote, Minnesota, USA) with Vcheck^®^ canine progesterone (Bionote, Minnesota, USA). This allowed a comparative analysis using different assessment methods for a more accurate investigation. The study maintained strict adherence to the manufacturer’s recommendations for laboratory and point-of-care assessment. This commitment has ensured the reliability, accuracy, and validity of the obtained progesterone data and provided a basis for credible findings and conclusions.

### Statistical analysis

In this study, we conducted a comprehensive analysis of serum progesterone concentration from the collected samples, encompassing the calculation of key statistical parameters, including the mean, standard deviation (SD), 95% confidence interval (CI), minimum (Min), and maximum (Max) value. Rigorous quantification was meticulously conducted across the significant phases of the bitch’s reproductive cycle, including proestrus, LH peak, pre-ovulation, ovulation, post-ovulation, and all stages. A paired t-test was used to assess the presence of a significant difference between CMIA and Vcheck^®^ means. All analyses were rigorously conducted utilizing the free trial version of XLSTAT in Microsoft Excel Home and Student Edition (WA, USA) (https://www.xlstat.com/en/download). The designated significance level was set at p < 0.05.

We conducted a comparative analysis with CMIA, a widely recognized reference method, to comprehensively evaluate the performance of Vcheck^®^. We employed Pearson’s correlation coefficient, a robust statistical tool, to assess the precision of both methods. To provide a more comprehensive view, we utilized Lin’s concordance correlation coefficient, which is known for its ability to rigorously evaluate agreement, to determine the agreement level between Vcheck^®^ and CMIA. It should be noted that perfect concordance is represented by a value of 1, indicating a perfect match between the two methods. In our evaluation, we also considered the bias correction factor as a critical measure of accuracy. Passing-Bablok regression and Bland-Altman analyses were performed to enhance further our understanding and comparison of Vcheck^®^ and CMIA values. These methods provide valuable insights into any systematic differences between the two techniques. These visuals vividly illustrate the outcomes of our analyses, making complex data more accessible and informative to readers.

## Results

Table-1 provides a comprehensive overview of the means, SD, 95% CI, and range of serum progesterone concentrations. These values were determined through measurements taken during various phases, including early proestrus, LH peak, pre-ovulation, ovulation, post-ovulation, and all bitch periods. This detailed presentation enables us to understand the distribution of progesterone levels in different phases.

**Table-1:** Mean, SD, 95% CI, Min, and Max value for serum progesterone concentration with quantification using the CMIA and Vcheck^®^ for estimates during the early proestrus, LH peak, pre-ovulation, ovulation, post-ovulation and all periods of the bitch.

Period	CMIA			Vcheck^®^		
	
	Mean ± SD	95% CI	Min-Max	Mean ± SD	95% CI	Min-Max
Proestrus	1.24 ± 0.37	1.11–1.38	1.00–1.98	1.36 ± 0.54	1.16–1.56	1.00–2.81
LH peak	2.65 ± 0.36^a^	2.35–2.95	2.17–2.98	1.88 ± 0.55^b^	1.42–2.33	1.00–2.73
Pre-ovulation	3.71 ± 0.52	3.31–4.10	3.00–4.44	3.35 ± 1.53	2.17–4.53	1.77–7.01
Ovulation	6.79 ± 1.32^a^	6.31–7.27	5.09–9.54	5.36 ± 2.48^b^	4.47–6.25	1.00–10.60
Post-ovulation	16.47 ± 7.18^a^	12.32–20.61	10.11–30.00	11.7 ± 5.90^b^	10.26–14.85	5.77–21.27
All period	5.75 ± 5.79^a^	4.57–6.94	1.00–30.00	4.50 ± 4.39^b^	3.60–5.40	1.00–21.27

^a, b^values within the same row with different superscripts mean statistically significant difference (p < 0.05). SD=Standard deviation, CI=Confidence interval, Min=minimum, Max=maximum, CMIA=Chemiluminescence microparticle immunoassay

**Figure-1:**
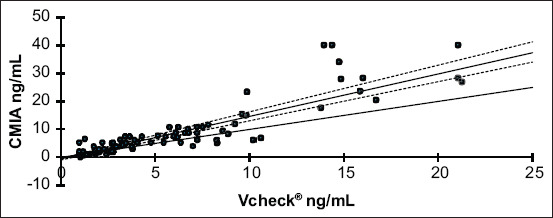
Passing-Bablok regression plot depicting serum progesterone measurements by Vcheck^®^ against the chemiluminescence microparticle immunoassay serum progesterone measurements. The thin line is the identity line. The thick line represents the regression line ant the dotted lines represent its 95% confidence interval.

We found an intriguing finding when we compared these values: The average values of all samples obtained using Vcheck^®^ were significantly lower than those obtained using CMIA, with an average difference of 1.26 ng/mL (Table-2 and Figure-1). This difference is not only statistically significant but also significantly impacts the accuracy and precision of the measurements.

**Table-2:** Measure of agreement: Concordance correlation coefficient, Pearsons’ correlation coefficient, ant bias correction factor.

95% limits of agreement (Bland and Altman)			Lin’s concordance correlation coefficient	95% confidence interval		Pearsons’ correlation coefficient	Bias correction factor
	
Average difference	Lower	Upper		Lower	Upper		
1.26	−3.16	5.68	0.877	0.834	0.910	0.939	0.935

**Figure-2:**
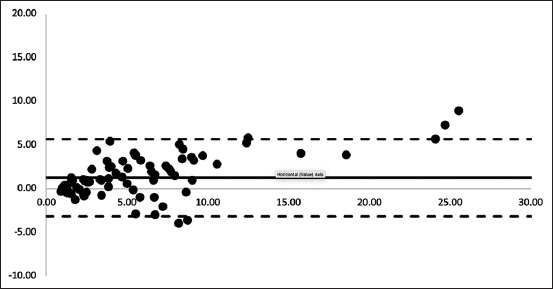
Bland-Altman plot depicting serum progesterone measurements by the Vcheck^®^ against the chemiluminescence microparticle immunoassay serum progesterone measurements. The black thick line represents the mean difference (bias) and the black dotted line represents the 95% confidence interval.

The bias correction factor, an essential component in our evaluation, was measured at 0.935, which was nearly 1.00. This proximity to 1.00 indicates that the best-fit line corresponds closely to the perfect line of agreement. This finding strengthens our confidence in measurement accuracy (Table-2 and Figure-2).

In addition, Pearson’s correlation coefficient reached a value of 0.939, confirming the very good precision of our measurements (Table-2). This high level of precision enhances the credibility of our findings.

In addition, Lin’s concordance correlation coefficient was found to be 0.877, indicating fair overall agreement between the Vcheck^®^ and CMIA techniques (Table-2 and Figure-2). This metric adds another layer of validation to our assessment, demonstrating a fair level of agreement between the two techniques.

## Discussion

An accurate and impartial assessment of progesterone concentrations is essential when assessing the reproductive status of bitches, in particular when determining the optimal breeding time and when forecasting or supervising the parturition date. RIA methods have been considered the gold standard for quantifying levels in bitches over the years [7, 22–24]. However, in 2014, the CLIA method was widely accepted and applied for progesterone measurement [25–27]. CMIA, an advanced adaptation of CLIA, is currently employed by veterinary reference laboratories in Thailand [18]. It’s important to note that both CLIA and CMIM have drawbacks, as they require several hours to several days for processing, depending on the laboratory’s location. On the other hand, point-of-care or in-house measurement of progesterone is gaining popularity among veterinary practitioners because of its simplicity, convenience, and rapid delivery.

In this study, serum handling differed based on the analyzers used: half of the harvested serum underwent analysis with the CMIA analyzer, whereas the remaining half was frozen and stored at –20°C before Vcheck^®^ testing. This divergence addressed specific aspects of the study’s objectives. Bolelli *et al*. [28] reported a decrease in progesterone levels after long-term storage at –70°C, potentially attributed to molecular modification or interference by the cryotube material. This insight is pertinent to our study because it raises the question of long-term storage effects on analytes. Conversely, Key *et al*. [29] reported apparently normal progesterone levels in sera stored at –20°C for up to 7 years. This finding contrasts with Bolelli *et al*.’s observations and adds complexity to our understanding of storage effects on progesterone levels. Volkmann [27] investigated the effects of anticoagulants, storage time, temperature, and assay methods on blood progesterone concentrations in dogs, expanding our understanding of the variables influencing progesterone measurements. The study’s outcomes yielded crucial insights: (i) RIA measurements indicated significantly higher sP4 concentration than CLIA; (ii) initial refrigeration of whole blood within 2 h post-collection notably decreased serum progesterone concentration; (iii) storage temperature of whole blood for up to 5 h had no apparent impact on progesterone concentration in heparinized plasma; and (iv) refrigeration of whole, clotted blood had no effect on serum progesterone concentration, given samples were at room temperature (25°C) for the first 2 h after collection. These findings are consistent with the main focus of this study on understanding the factors influencing blood progesterone levels in canines. To improve coherence, the findings of this study directly inform the primary focus on understanding the effects of various factors on blood progesterone levels in canine subjects. Therefore, in this study, all samples were promptly separated within 2 h after whole blood collection to obtain serum, ensuring accurate analysis. To maintain sample integrity and stability before Vcheck^®^ testing, rapid separation and subsequent storage at –20°C were conducted to minimize any potential degradation of analytes.

The rapid measurement of serum progesterone levels is of the utmost importance as it allows accurate diagnoses and informed clinical decisions, especially in situations such as breeding or the careful planning of cesarean section. In view of the diverse requirements for precise diagnostics and well-considered decision-making, veterinarians consider commercial immunological analyzers indispensable. Several researchers have reported the accuracy of other commercial point-of-care immunologic analyzers compared with gold-standard methods, such as RIA, LC-MS, and computed tomography (CLIA) [5, 18–20, 30]. However, the present study is the first to report the use of the Vcheck^®^ analyzer, providing novel insights into its accuracy and application. In conclusion, this study confirms the reliability of the Vcheck^®^ analyzer for the swift measurement of serum progesterone levels in bitches. The Pearson’s correlation coefficient between Vcheck^®^ and CMIA exceeded the significance threshold of 0.09 (Table-2). These results undeniably indicate a strong and significant relationship [31]. This discovery offers compelling proof of the inherent accuracy of serum progesterone concentration measurements obtained using Vcheck^®^. Table-1 presents the mean, SD, 95% CI, and range of serum progesterone concentration. This allows for a detailed comparison between Vcheck^®^ and CMIA in estimating these values across different phases of the female dog’s reproductive cycle, including proestrus, LH peak, pre-ovulation, and ovulation and post-ovulation. This table exhibits distinct sets of values, designated with l lowercase letters and meticulously organized in rows corresponding to the Vcheck^®^ analyzer. Each value marked with a specific superscript indicates a statistically significant difference (p < 0.05) when compared to the reference CMIA values. On the other hand, the uppercase letter values, similarly marked with their own set of superscripts, show no statistically significant difference (p > 0.05). The absence of a significant difference signifies the comparability of these results to those obtained through CMIA. Notably, when using the Vcheck^®^ analyzer, no significant difference was observed during the proestrus and pre-ovulation phases of the meticulously estrous cycle.

Our findings have the potential to inform the effective management of optimal breeding times, drawing implications from the 95% CI and range (min–max) of serum progesterone results (Table-1). Moreover, this study paves the way for future research endeavors, especially exploring specific enhancements in the assessment of serum progesterone. These improvements strengthen veterinary practices and advance reproductive management by providing more accurate and reliable tools. In accordance with this objective, the guidelines presented here have been carefully formulated. These guidelines were devised by aligning with established CMIA guidelines and adapting them through the incorporation of range and 95% CI derived from each set of Vcheck^®^ results (Table-3).

**Table-3:** Reference or guideline for progesterone interpretation using Vcheck^®^ in heat or apparent reproductively quiescent bitches.

Progesterone by CMIA (ng/mL)	Progesterone by Vcheck^®^ (ng/mL)	Likely events	Suggestion

	Min-max (95% confidence interval)		
<2	1.00–2.81 (1.16–1.56)	Anestrus, proestrus, and pre-LH surge	• Confirm heat or proestrus by physical examination or vaginal cytology. • Retest in 3 days
2.00–2.99	1.00–2.73 (1.42–2.33)	LH surge	• Retest in 2 days to confirm continued rise in progesterone. • Aim for breeding 4–7 days.
3.00–4.99	1.77–7.01 (2.17–4.53)	Post-LH surge, pre-ovulation	• Retest in 1–2 days to confirm continued rise in progesterone. • Aim for breeding 3–5 days.
5.00–9.99	1.00–10.60 (4.47–6.25)	At or near ovulation	• Retest in 1 days to confirm continued rise in progesterone. • Aim for breeding 2–4 days.
>10	5.77–21.27 (10.26–14.85)	Post-ovulation, Oocyte maturation, in fertilizable period	• Aim for breeding on this day and for another 2 days hereafter.

Min=minimum, Max=maximum, CMIA=Chemiluminescence microparticle immunoassay

The aim of the present study was to confirm the validity of the Vcheck^®^ analyzer for measuring serum progesterone levels in bitches, which is a crucial factor in assessing their reproductive status. Serum progesterone concentrations usually show a sharp increase (spike) at ovulation following a slow increase in proestrus, peak LH, and pre-ovulation. This pattern is pivotal in understanding the reproductive cycle. More importantly, this study demonstrated that the Vcheck^®^ analyzer was as accurate as CMIA techniques in detecting serum progesterone concentrations in bitches, providing a reliable tool for veterinarians. One of the advantages of the Vcheck^®^ analyzer is its shorter turnaround time for obtaining results, which can expedite decision-making in breeding management. In addition, our investigation involved a comprehensive assessment of 59 bitches to determine the ovulation day and optimal breeding date prediction. This examination encompassed the utilization of both the CMIA and Vcheck^®^ methods during the proestrus and estrus periods before breeding. These animals underwent artificial insemination at or close to the ovulation phase 2–4 days later. The results were very promising, 51 out of 59 dogs were successfully conceived, resulting in an 86.44% pregnancy rate. In addition, parturition was achieved in 48 out of 59 dogs, resulting in 81.36% parturition rate. These findings underscore the efficacy of the CMIA and Vcheck^®^ analyzer and their pivotal role in enhancing breeding and parturition outcomes. The shorter turnaround time offered by the Vcheck^®^ analyzer facilitates decision-making in breeding management, which benefits both veterinarians and breeders.

## Conclusion

The Vcheck^®^ analyzer provides a rapid assessment of serum progesterone concentration in bitches, with results comparable to those obtained using the CMIA method. However, when considering the use of the Vcheck^®^ analyzer, it is advisable to interpret the results carefully and follow the interpretation guidelines given in Table-3. In conclusion, Vcheck^®^ provides a reliable and convenient option for veterinarian practitioners to measure canine progesterone levels in a clinical/hospital setting.

## Authors’ contributions

SW, TS, SA, WP, and SR: Study conception and design, conducted the study, and analyzed the data. SW and SR: Sample preparation. SW, TS, and SR: Drafted the manuscript. All authors have read, reviewed, and approved the final manuscript.
